# RNA atlas and competing endogenous RNA regulation in tissue-derived exosomes from luminal B and triple-negative breast cancer patients

**DOI:** 10.3389/fonc.2023.1113115

**Published:** 2023-07-07

**Authors:** Ji Wang, Xianyu Zhang, Zilong You, Yuhuan Meng, Xijie Fan, Guangdong Qiao, Da Pang

**Affiliations:** ^1^ Medical Translational Research Institute, Guangzhou KingMed Center for Clinical Laboratory Co., Ltd, Guangzhou, China; ^2^ Department of Breast Surgery, Yantai Yuhuangding Hospital, Yantai, China; ^3^ Department of Breast Surgery, Harbin Medical University Cancer Hospital, Harbin, China

**Keywords:** luminal B, triple-negative breast cancer, tissue-derived exosome, competitive endogenous RNA, RAS–MAPK pathway

## Abstract

**Background:**

Luminal B and triple-negative breast cancer (TNBC) are malignant subtypes of breast cancer (BC), which can be attributed to the multifaceted roles of tissue-derived exosomes (T-exos). Competing endogenous RNA (ceRNA) networks can regulate gene expression post-transcriptionally.

**Methods:**

RNAs in T-exos from luminal B BC (*n*=8) and TNBC (*n*=8) patients were compared with those from persons with benign breast disease (*n*=8). The differentially expressed (DE) mRNA, microRNA (miRNA), and long noncoding RNA (lncRNA) target genes were annotated using Gene Ontology (GO) and Kyoto Encyclopedia of Genes and Genomes (KEGG) to reveal the relevant biological processes.The ceRNA networks were constructed to show distinct regulation, and the mRNAs involved were annotated. The miRNAs involved in the ceRNA networks were screened with the Kaplan–Meier Plotter database to identify dysregulated ceRNAs with prognostic power.

**Results:**

In total, 802 DE mRNAs, 441 DE lncRNAs, and 104 DE miRNAs were identified in luminal B BC T-exos, while 1699 DE mRNAs, 590 DE lncRNAs, and 277 DE miRNAs were identified in TNBC T-exos. Gene annotation revealed that the RAS–MAPK pathway was the primary biological process in luminal B BC T-exos, while endocrine system development and growth were the main processes in TNBC T-exos. Survival analysis established seven survival-related lncRNA/miRNA/mRNA regulations in luminal B BC T-exos, and nineteen survival-related lncRNA/miRNA/mRNA regulations in TNBC T-exos.

**Conclusion:**

In addition to survival-related ceRNA regulations, ceRNA regulation of RAS–MAPK in luminal B and endocrine system development and growth regulation in TNBC might contribute to the tumorigenesis of BC.

## Introduction

Breast cancer (BC) arises in the epithelium of the ducts or lobules of the breast glandular tissue and is the most common cancer among women. According to the GLOBOCAN 2020 data, BC is the fifth most common cause of cancer-related deaths in the whole population (1). Dysregulation of gene expression is a hallmark of BC with aggressive biological behavior (2, 3). Gene expression profiling greatly facilitates the diagnosis and treatment of BC patients by identifying four molecular subtypes (1): luminal A, presence of the estrogen receptor (ER) and/or the progesterone receptor (PR), absence of human epidermal growth factor receptor 2 (HER2); (2) luminal B, ER positive, PR negative, and/or HER2 positive; (3) HER2-enriched; (4) triple-negative breast cancer (TNBC), ER negative, PR negative, and HER2 negative (4). On the other hand, breast cancer cell lines can be genetically and epigenetically categorized into five subtypes, i. e., luminal A, luminal B, HER2 positive, triple negative A, and triple negative B (5). Among these subtypes, luminal B requires more aggressive treatment, and TNBC is frequently of a high grade and has a high rate of recurrence (6).

A rapid and efficient way to regulate gene expression is *via* the regulation of mRNA stability through a post-transcriptional mechanism. Among the various regulation mechanisms, the competing endogenous RNA (ceRNA) network plays an essential role in the carcinogenesis and progression of BC (7). Long noncoding RNAs (lncRNAs) can function as ceRNAs to sequester microRNAs (miRNAs) and prevent the downregulation of target mRNAs. Recently, several ceRNA networks have been determined to have fundamental roles in BC development, thus establishing hallmarks of this disease (8). However, limited research has been performed to decipher the subtype-specific ceRNA networks.

As a lipid-bilayer membrane secreted by parent cells, exosomes can transfer lipids, proteins, and nucleic acids to mediate intercellular communication and signal transduction between parent and recipient cells (9). Emerging evidence demonstrates that exosomes in the tumor microenvironment promote the reprogramming and recruitment of relevant constituents (10, 11). Great attention has been paid to tissue-derived exosomes to identify the tissue-specific microenvironment background, which can reflect the pathophysiological characteristics of the tumor microenvironment (12). Thus, the relevant ceRNA network in exosomes derived from BC tissue will open a new window to decipher the hidden aspects of different subtypes of BC.

**Figure 1:**
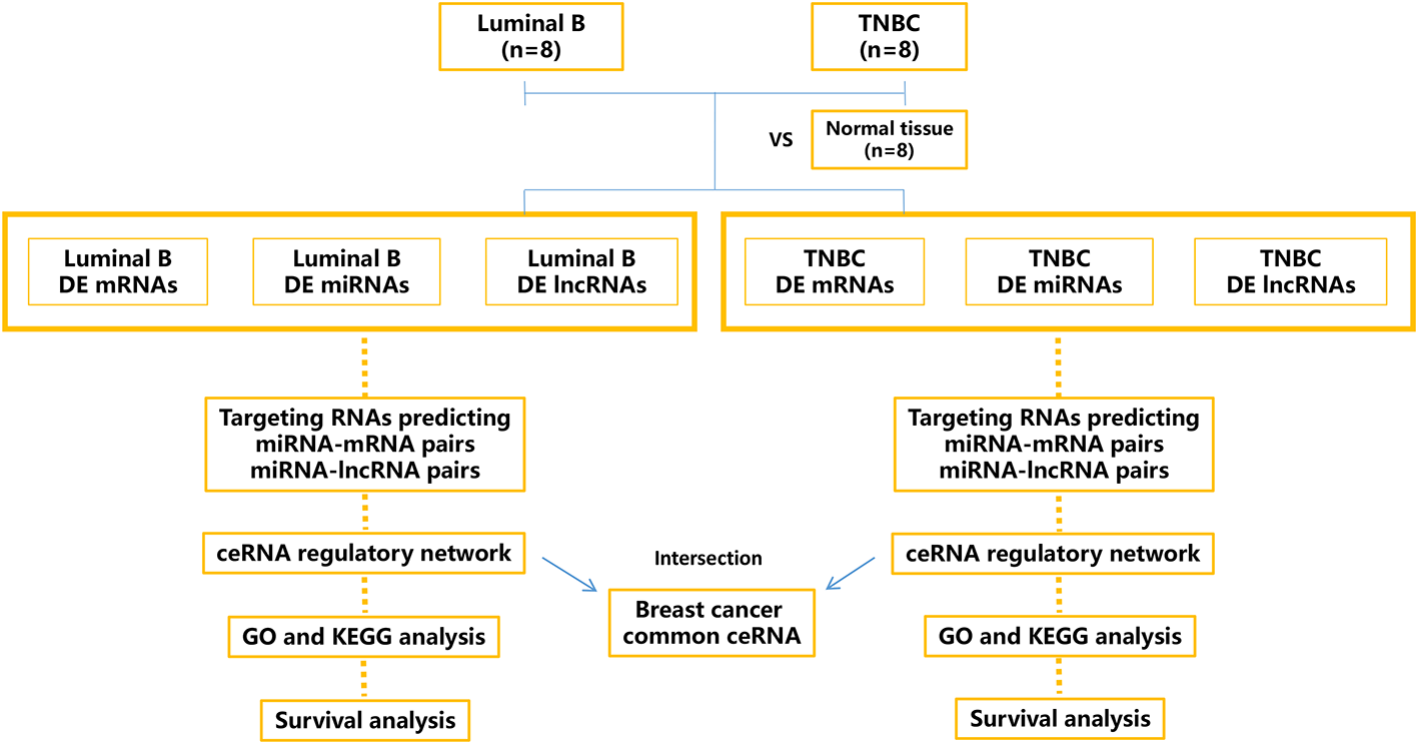
Schematic outline of the workflow of this study. Tissue-derived exosomes from luminal B breast cancer and triple-negative breast cancer (TNBC) were sequenced, and the differentially expressed (DE) mRNAs, miRNAs, and lncRNAs were calculated and annotated. The relevant ceRNA networks (lncRNA/miRNA/mRNA) of luminal B breast cancer and TNBC were constructed, and the intersection ceRNA network was identified.

This study aimed to construct the dysregulated RNA [long intergenic noncoding RNA (lncRNA), miRNA, and mRNA] atlas and the ceRNA network based on lncRNA/miRNA/mRNA regulation **Figure 1**. Our data suggest that ceRNA regulatory profiles could be valuable predictive parameters for patient survival. Further investigation might greatly promote understanding of the post-transcriptional mechanism underlying different subtypes of BC.

## Materials and methods

### Clinical specimens

BC tissues were obtained from patients undergoing primary resection at Yantai Yuhuangding Hospital. Luminal B BC (*n*=8) was diagnosed by the expression of ER and/or PR and the overexpression of HER2. TNBC (*n*=8) was diagnosed by immunohistochemistry and characterized by the lack of expression of ER, PR, and HER2. Individuals with benign breast disease were enrolled in the control group (*n*=8). Written informed consent was obtained from each participant, and the study was approved by the Ethics Board of Yantai Yuhuangding Hospital.

### Separation of tissue-derived exosomes

BC tissue and normal tissue were dissociated with a Human Tumor Dissociation Kit, according to the manufacturer’s instructions (Miltenyi Biotec, cat. no. 130-095-929). The dissociated tissue was gently filtered through a 70-μm filter twice to remove residual tissues. The suspension was sequentially centrifuged (300×*g* at 4°C for 10 min and 2000×*g* at 4°C for 10 min) to obtain the cell-free supernatant, which was further centrifuged (10,000×*g* at 4°C for 20 min), ultracentrifuged (150,000×*g* at 4°C for 2 h), resuspended in 1 mL of phosphate-buffered saline, and further purified using Exosupur^®^ columns (Echobiotech, China). Fractions were concentrated to 200 μL by 100-kDa molecular weight cut-off Amicon^®^ Ultra spin filters (Merck, Germany). The size and morphology of the exosomes were quantified with nanoparticle tracking analysis and transmission electron microscopy. The markers of exosomes (CD9, CD63, Tsg101, and calnexin) were detected by western blot analyses.

### Library preparation and sequencing

Total RNA in tissue-derived exosomes was extracted with an miRNeasy Kit (Qiagen, Frederick, MD, USA). For mRNA and lncRNA library construction, an EpicentreRibo-Zero™ rRNA Removal Kit (Epicentre Biotechnologies, Madison, WI, USA) was first utilized to remove ribosomal RNA, and a NEBNext^®^ Ultra™ Directional RNA Library Prep Kit was applied to generate library fragments, which were further amplified on an Agilent Bioanalyzer 2100. In addition, a QIAseq miRNA Library Kit was utilized to construct the miRNA library. Reverse transcription primers ligated with unique molecular indices were applied to quantify the relative miRNA expression levels on an Agilent Bioanalyzer 2100. Libraries were sequenced on an Illumina NovaSeq6000 system (Illumina, San Diego).

### lncRNA and mRNA data quantification and differential expression analysis

Raw FASTQ data were processed with in-house Perl scripts to obtain the paired-end clean reads, which were aligned to GRCh38. Mapped reads were further utilized for gene expression quantification and differential analysis. Coding potential and lncRNA target gene prediction were performed as described previously (13). Coding-Non-Coding-Index (V2), Pfam Scan (v1.3), Coding Potential Calculator (0.9-r2), and Phylogenetic Codon Substitution Frequency (v20121028) with default parameters were utilized to predict the coding potential. Cis-acting lncRNAs were screened and located within the 100-kb downstream and upstream regions of the target genes. Additionally, the correlation between the differentially expressed (DE) lncRNAs and potential trans-acting genes were calculated (Pearson correlation, *r*>0.95). Differential expression analysis was performed with the Mann–Whitney U test (FPKM>5, *P*-value<0.05, and fold change>1.5).

### miRNA data quantification and differential expression analysis

FastX software was used to obtain clean reads, which were further mapped to the GtRNAdb database, Repbase database, and Rfam database using TopHat v2.0.981 and aligned to known mature human miRNA sequences downloaded from the Human Reference Genome (GRCh38). mirDeep2 (v2.0.0.5) was applied to predict novel miRNAs and candidate target genes. The Mann–Whitney U test was used for differential expression analysis (FPKM>5, *P*-value<0.05, and fold change>1.5).

### Gene ontology and Kyoto encyclopedia of genes and genomes pathway annotation analyses

DE genes and predicted target genes of the DE lncRNAs and miRNAs were annotated with KOBAS software (14) or Metascape (15) based on GO and KEGG analyses. Corrected *P*-values<0.05 was considered to be significantly enriched.

### Construction of the ceRNA network

Predicted miRNA–mRNA interactions were retrieved from miRanda, Targetscan, and miRWalk. Moreover, miRNA–lncRNA interactions were predicted with miRanda and PITA. Finally, the interaction information was integrated as follows: (1) negative expression correlation; (2) Pearson correlation coefficient>0.5.

### Overall survival analysis

The miRpower, a web database to screen overall survival-associated miRNAs (2,178 BC patients), was utilized in this study (16). Kaplan–Meier analysis was performed to screen the miRNAs involved in the ceRNA network that was able to predict patient survival.

## Results

### Dataset acquisition and identification of dysregulated RNAs

Tissue exosomes derived from luminal B BC and TNBC were analyzed for their size, morphology, and specific marker expression (**Supplementary Figure 1**). RNA sequencing data from 16 BC samples (8 luminal B BC samples and 8 TNBC samples) and 8 normal breast samples were utilized to explore the potential risk of DE RNAs. A total of 802 DE mRNAs (525 upregulated, 277 downregulated), 441 DE lncRNAs (241 upregulated, 200 downregulated), and 104 DE miRNAs (69 upregulated, 35 downregulated) in luminal B BC tissues compared with normal tissues, as well as 1,699 DE mRNAs (1040 upregulated, 659 downregulated), 590 DE lncRNAs (393 upregulated, 197 downregulated), and 277 DE miRNAs (213 upregulated, 64 downregulated) in TNBC compared with normal tissues (**Supplementary Table 1**) were found. There were fewer of the three types of DE RNAs in luminal B BC than in TNBC, indicating less heterogeneity in the former. In total, 378 shared dysregulated mRNAs, 48 shared dysregulated miRNAs, and 120 shared dysregulated lncRNAs were concordantly changed in luminal B BC and TNBC (**Supplementary Table 2**). Notably, downregulated ER1 and PR were observed in tissue exosomes derived from TNBC tissues, which was consistent with previous studies (17).

**Figure 2:**
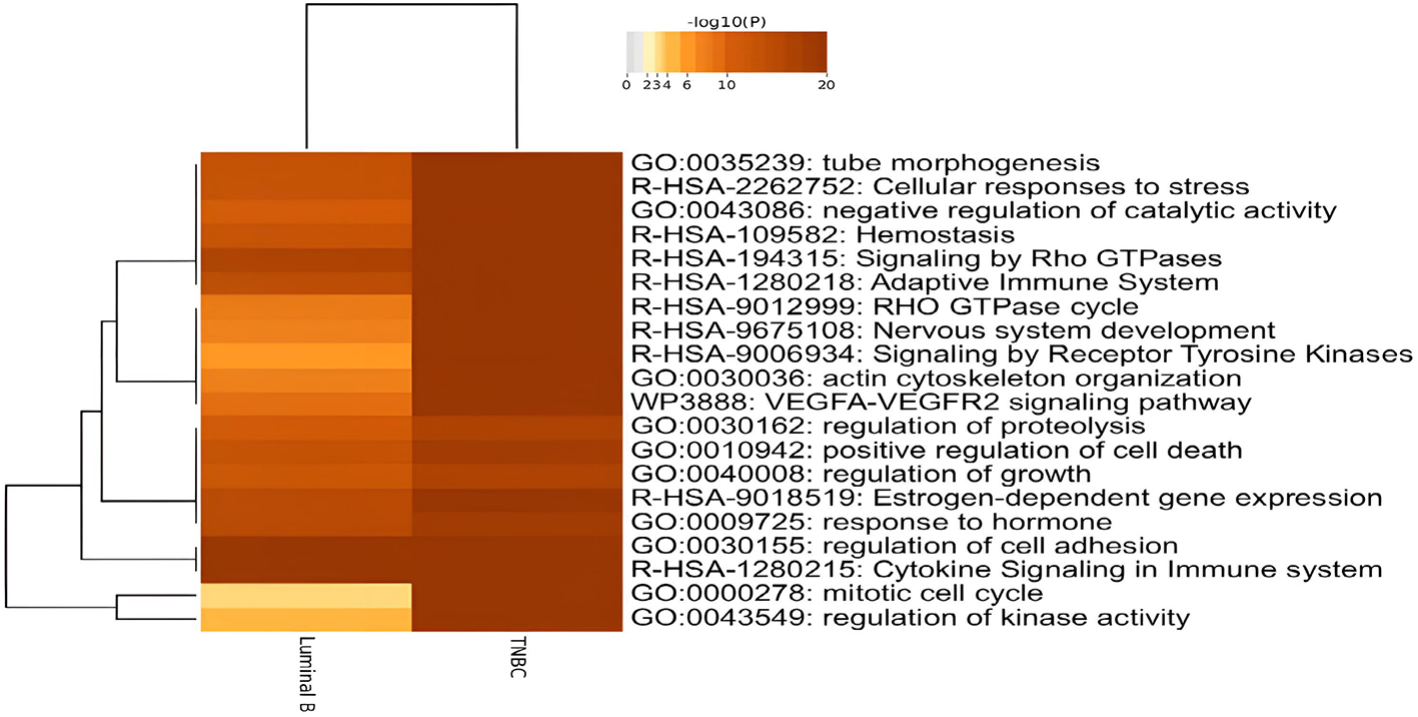
Dysregulated biological functions of differentially expressed genes (DEGs) in tissue exosomes derived from luminal B breast cancer and triple-negative breast cancer (TNBC) through enrichment analyses. The DEGs were submitted to Metascape, and the enriched terms were presented with Heatmap.

The DE genes identified in the tissue exosomes derived from luminal B BC and TNBC were annotated with Metascape (**Figure 2**). As expected, estrogen-dependent gene expression (R-HSA-9018519) and response to hormone (GO:0009725) were annotated in both luminal B BC and TNBC. On the other hand, signaling by Rho GTPases (R-HSA-194315), the RHO GTPase cycle (R-HSA-9012999), and the VEGFA-VEGFR2 pathway (WP3888) were annotated as significant signals that might contribute to the development of both subtypes. In contrast, no BC subtype-specific annotation was found.

**Figure 3:**
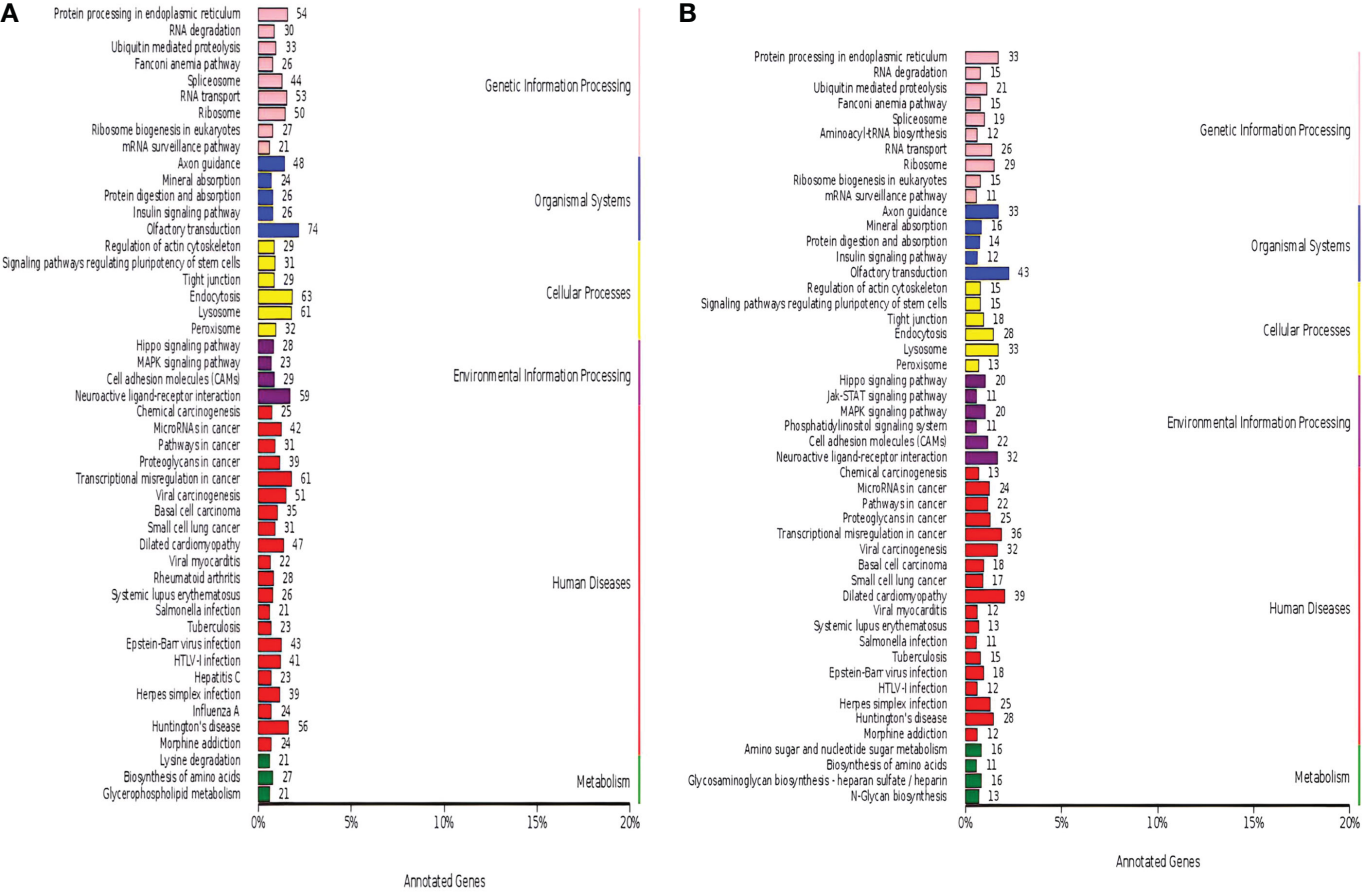
Dysregulated Kyoto Encyclopedia of Genes and Genomes (KEGG) annotation of differentially expressed (DE) miRNAs in tissue exosomes derived from luminal B breast cancer and triple-negative breast cancer (TNBC). The DE miRNA-predicted target genes in tissue exosomes derived from luminal B breast cancer **(A)** and TNBC **(B)** were annotated with KOBAS software. Corrected *P*-values <0.05 were considered to be significantly enriched.

The miRNA-targeted gene annotation analysis demonstrated that the Hippo signaling pathway (ko04390), cell adhesion molecules (ko04514), the MAPK signaling pathway (ko04010), and the neuroactive ligand-receptor interaction (ko04080) were common annotations in both luminal B BC (**Figure 3A**) and TNBC (**Figure 3B**); while the phosphatidylinositol signaling system (ko04070) and the JAK–STAT signaling pathway (ko04630) were TNBC-specific pathways according to KEGG analysis. Among the DE miRNAs identified, only hsa-miR-184, hsa-miR-206, hsa-miR-429, hsa-miR-663a, and hsa-miR-760 related to the luminal B subtype were breast neoplasm-associated miRNAs as retrieved in the Human MicroRNA Disease Database. However, no breast neoplasm-associated miRNAs were found in the TNBC subtype.

**Figure 4:**
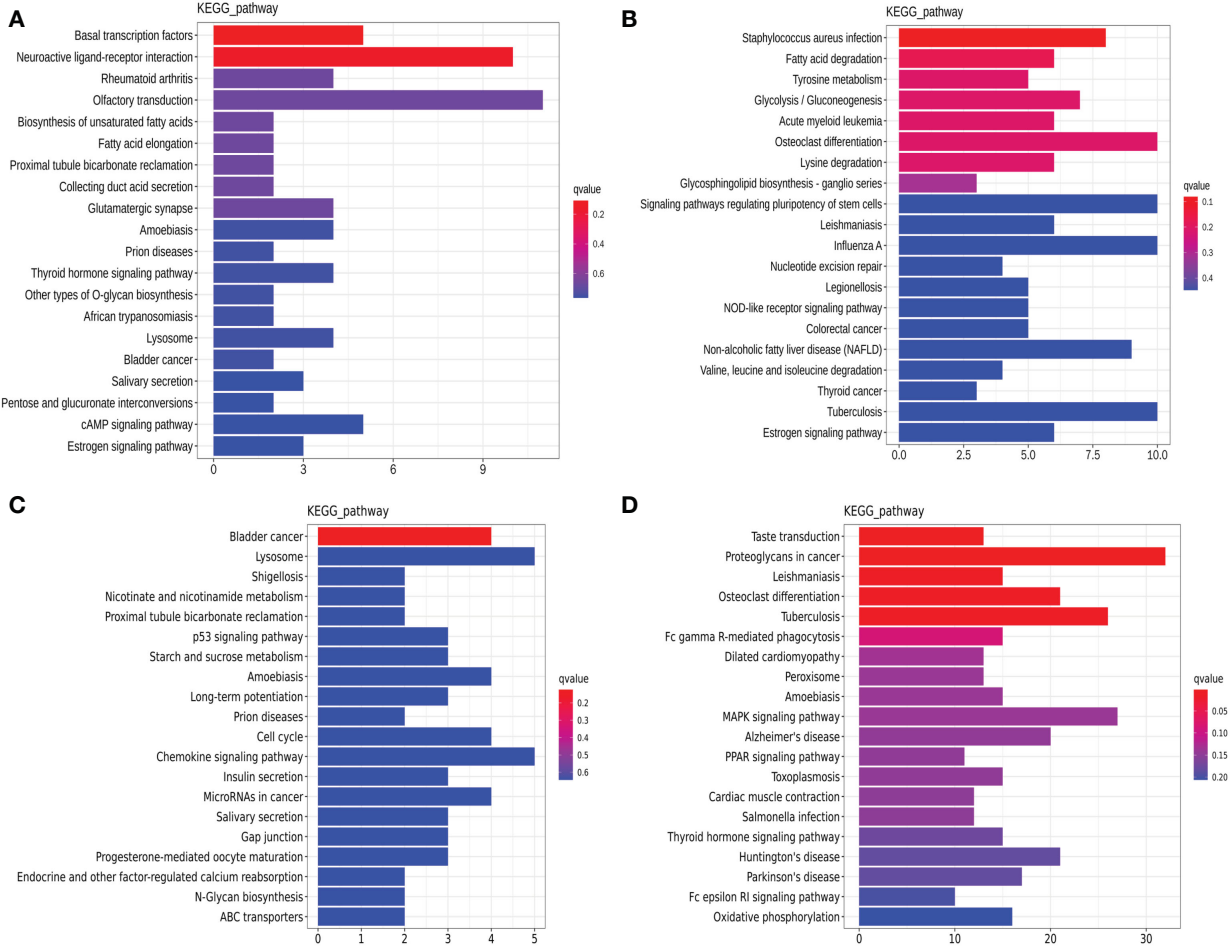
Dysregulated Kyoto Encyclopedia of Genes and Genomes (KEGG) annotation of differentially expressed (DE) lncRNAs in tissue exosomes derived from luminal B breast cancer and triple-negative breast cancer (TNBC). The DE trans-acting lncRNA-predicted target genes **(A, C)** and cis-acting lncRNA-predicted target genes **(B, D)** in tissue exosomes derived from luminal B breast cancer **(A, B)** and TNBC **(C, D)** were annotated with KOBAS software. Corrected *P*-values <0.05 were considered to be significantly enriched.

The lncRNA-targeted gene analysis demonstrated that the thyroid hormone signaling pathway (hsa04919, **Figures 4A, D**) and the osteoclast differentiation signaling pathway (hsa04380, **Figures 4B, D**) were the common pathways annotated in both the luminal B and TNBC subtypes. On the other hand, the estrogen signaling pathway (hsa04915, **Figure 4A**) was annotated in luminal B BC, and the p53 (hsa04115, **Figure 4C**), PPAR (hsa03320, **Figure 4D**), and MAPK signaling pathways (hsa04010, **Figure 4D**) were annotated in TNBC. It is worth noting that few genes overlapped between the miRNA-predicted gene intersection and the lncRNA-predicted gene intersection, indicating that ceRNA regulation might be the main type of regulation. In the following analysis, the possible ceRNA regulation was deciphered in luminal B BC and TNBC.

### ceRNA network in tissue exosomes derived from the luminal B subtype

**Figure 5:**
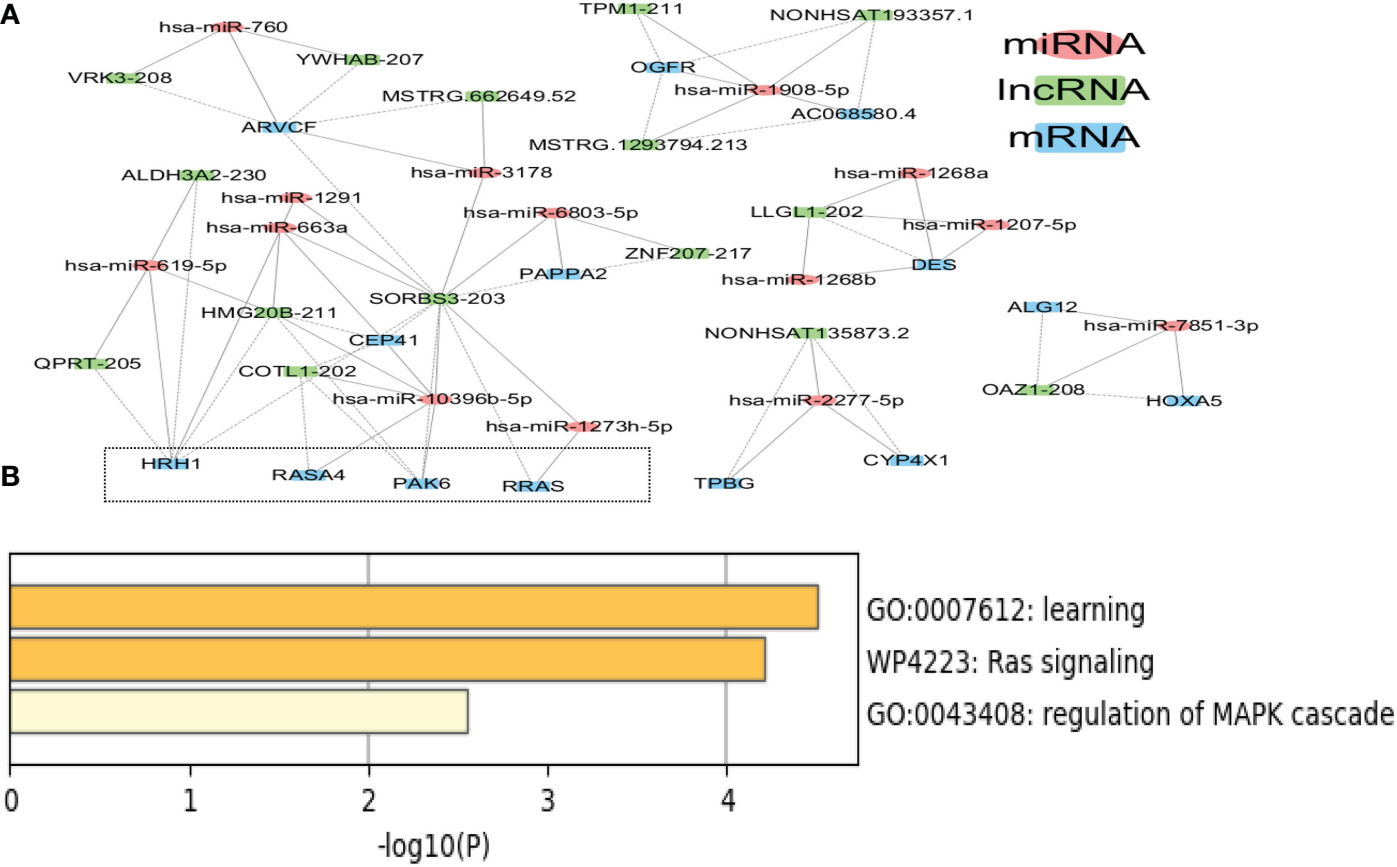
Competing endogenous RNA (ceRNA) network in tissue exosomes derived from luminal B breast cancer. **(A)** The ceRNA network of tissue exosomes derived from luminal B breast cancer based on lncRNA/miRNA/mRNA. **(B)** The mRNAs in the ceRNA network were annotated with Metascape (with default parameters).

The ceRNA network in tissue exosomes derived from the luminal B subtype was constructed (**Figure 5A**), and 46 nodes (16 lncRNAs, 15 miRNAs, and 15 mRNAs) and 71 interactions were found. To decipher the potential biological functions of the ceRNA network, the mRNAs involved were annotated with Metascape (**Figure 5B**) as follows: the RAS-related protein R-RAS (RRAS), RAS P21 protein activator 4 (RASA4), and P21 activated kinase 6 (PAK6) genes were annotated into RAS signaling (WikiPathways, WP4223); RRAS, trophoblast glycoprotein (TPBG), and PAK6 were annotated into the regulation of the MAPK cascade (GO Biological Processes, GO:0043408); and histamine receptor 1 (HRH1), TPBG, and PAK6 were annotated into learning (GO Biological Processes, GO: GO:0007612). The relevant genes are framed with a dotted line in **Figure 5A**.

It is worth noting that hsa-miR-1273h-5p, hsa-miR-10396b-5p, and hsa-miR-2277-5p might regulate the RAS–MAPK pathway; among these miRNAs, hsa-miR-1273h and hsa-miR-2277 could be utilized to predict the survival of BC patients. In addition to hsa-miR-1273h and hsa-miR-2277, hsa-miR-1908, hsa-miR-1268, hsa-miR-7851, hsa-miR-6803, and hsa-miR-3178 involved in the ceRNA network could predict the overall survival of BC patients (**Supplementary Figure 2**), indicating that ceRNA regulation was related to the survival of BC patients with the luminal B subtype (**Table 1**).

**Table 1:** The potential ceRNAs in tissue exosomes derived from luminal B BC patients that predict patient survival.

lncRNA	miRNA	mRNA
MSTRG.1293794.213, NONHSAT193357.1	hsa-miR-1908-5p	OGFR, AC068580.4
LLGL1	hsa-miR-1268	DES
OAZ1	hsa-miR-7851-3p	ALG12, HOXA5
SORBS3	hsa-miR-1273h	RRAS
NONHSAT135873.2	hsa-miR-2277-5p	TPBG, CYP4X1
SORBS3, ZNF207	hsa-miR-6803-5p	PAPPA2
SORBS3, MSTRG.662649.52	hsa-miR-3178	ARVCF

On the other hand, based on the complex and topological centrality characteristics, the dysregulated RAS–MAPK pathway-related RNAs were not the hub node in the ceRNA network. In order to decipher the essentiality of the related RNAs, the hub RNAs (SORBS3, ARVCF, HMG20B, HRH1, and hsa-miR-10396b-5p, degree >5) in the ceRNA network in tissue exosomes derived from the luminal B subtype are provided in **Table 2**. Among the hubs, SORBS3 could co-activate ERα signaling to repress STAT3 signaling in hepatocellular carcinoma; however, the potential activation of ERα in luminal B BC requires further analysis. Altogether, these results indicate that tissue exosomes derived from the luminal B subtype might regulate the RAS–MAPK pathway and that ceRNA regulation might predict the survival of luminal B BC patients.

**Table 2:** The hub RNAs (degree>5) in the ceRNA network in tissue exosomes derived from luminal B BC and TNBC patients.

Luminal B			TNBC		
Hub	Degree	Expression	Hub	Degree	Expression
SORBS3-203	12	Down	PAK6	18	Down
ARVCF	6	Down	hsa-miR-10396b-5p	10	Up
HMG20B-211	6	Down	WBP2-207	10	Down
HRH1	6	Down	ZFYVE21-209	9	Down
hsa-miR-10396b-5p	6	Up	OAZ1-208	8	Up
			SORBS3-203	7	Down
			SNUPN	7	Down
			ANTXR1-204	7	Down
			ALG12	7	Down
			CYB5D2	7	Down
			hsa-miR-7851-3p	6	Up
			CALCOCO1	6	Down
			PAMR1	6	Down
			AP001931.2	6	Down

The RNAs labeled in red font were also the intersection ceRNAs listed in **Table 3**.

**Table 3:** The intersection ceRNAs in tissue exosomes derived from luminal B BC and TNBC patients.

Hub	Type	Luminal B	TNBC
COTL1-202	lncRNA	Down	Down
OAZ1-208	lncRNA	Down	Down
SORBS3-203	lncRNA	Down	Down
HMG20B-211	lncRNA	Down	Down
hsa-miR-10396b-5p	miRNA	Up	Up
hsa-miR-7851-3p	miRNA	Up	Up
ALG12	mRNA	Down	Down
HOXA5	mRNA	Down	Down
PAK6	mRNA	Down	Down

### ceRNA network in tissue exosomes derived from the TNBC subtype

**Figure 6:**
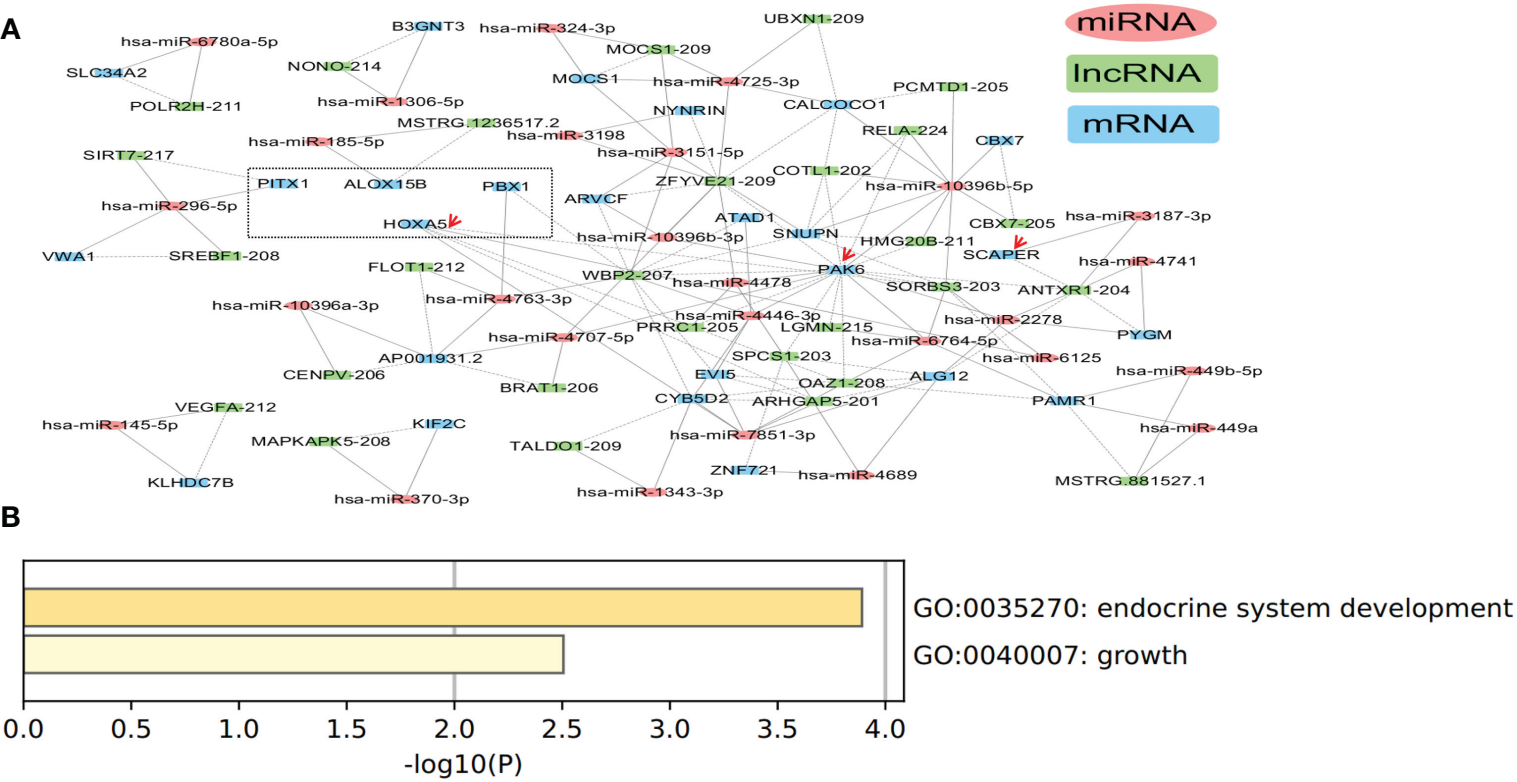
Competing endogenous RNA (ceRNA) network in tissue exosomes derived from triple-negative breast cancer (TNBC). **(A)** The ceRNA network of tissue exosomes derived from TNBC based on lncRNA/miRNA/mRNA. **(B)** The mRNA in the ceRNA network were annotated with Metascape (using default parameters).

The ceRNA network in tissue exosomes derived from the TNBC subtype was constructed, and 83 nodes (29 lncRNAs, 28 miRNAs, and 26 mRNAs) and 147 interactions were found (**Figure 6A**). Function annotation of the ceRNA network with involved mRNAs was performed with Metascape (**Figure 6B**). HOXA5, PBX1, PITX1, and ALOX15B (framed with a dotted line) were annotated into endocrine system development (GO Biological Process, GO:0035270). Meanwhile, HOXA5, SCAPER, and PAK6 (labeled with red arrows) were annotated into growth (GO Biological Process, GO:0040007), which might contribute to the high proliferation characteristics of TNBC. All of these annotations indicated that tissue exosomes derived from TNBC might regulate endocrine system (gland) development and growth-related biological processes.

Among the miRNAs identified in the ceRNA network, hsa-miR-7851-3p and hsa-miR-10396b-5p were the hub node (**Table 2**). On the other hand, 19 miRNAs (hsa-miR-4725, hsa-miR-449a, hsa-miR-3187, hsa-miR-6125, hsa-miR-4763, hsa-miR-2277, hsa-miR-4741, hsa-miR-4446, hsa-miR-1306, hsa-miR-2278, hsa-miR-185, hsa-miR-6764, hsa-miR-1343, hsa-miR-3151, hsa-miR-3198, hsa-miR-4478, hsa-miR-449b, hsa-miR-6780a, hsa-miR-4707, and hsa-miR-4689) were identified to have predictive value in BC (**Supplementary Figure 3**), and the potential ceRNAs that can predict patient survival are provided in **Table 4**. On the other hand, the mRNAs that have been demonstrated to be associated with patient survival are labeled in red. These data indicate that in addition to survival-related ceRNA regulation, endocrine system (gland) development and growth-related ceRNA regulation should be deciphered in the future.

**Table 4:** The potential ceRNAs in tissue exosomes derived from TNBC that can predict patient survival.

lncRNA	miRNA	mRNA	
UBXN1, ZFYVE21	hsa-miR-4725-3p	MOCS1, CALCOCO1	
MSTRG.881527.1	hsa-miR-449a	PAMR1
ANTXR1	hsa-miR-3187-3p	SCAPER
SORBS3	hsa-miR-6125	HOXA5
FLOT1	hsa-miR-4763	AP001931.2
ANTXR1	hsa-miR-4741	PYGM
WBP2	hsa-miR-4446	EVI5, PAK6, ATAD1, CYB5D2
NONO	hsa-miR-1306-5p	B3GNT3
ANTXR1	hsa-miR-2278	PYGM, ALG12, PAK6
MSTRG.1236517.2	hsa-miR-185-5p	ALOX15B
LGMN, SORBS3, OAZ1	hsa-miR-6764-5p	PAK6, PAMR1
TALDO1	hsa-miR-1343	CYB5D2
MOCS1, WBP2	hsa-miR-3151	ARVCF, SNUPN, MOCS1
ZFYVE21	hsa-miR-3198	NYNRIN
SPCS1, PRRC1, ZFYVE21	hsa-miR-4478	PAK6
MSTRG.881527.1	hsa-miR-449b-5p	PAMR1
POLR2H	hsa-miR-6780a-5p	SLC34A2
BRAT1	hsa-miR-4707	AP001931.2, PAK6
SPCS1	hsa-miR-4689	ALG12	

The mRNAs labeled in red font were found to be associated with survival (**Supplementary material**).

### The intersected ceRNAs in tissue exosomes derived from the luminal B and TNBC subtypes

**Figure 7:**
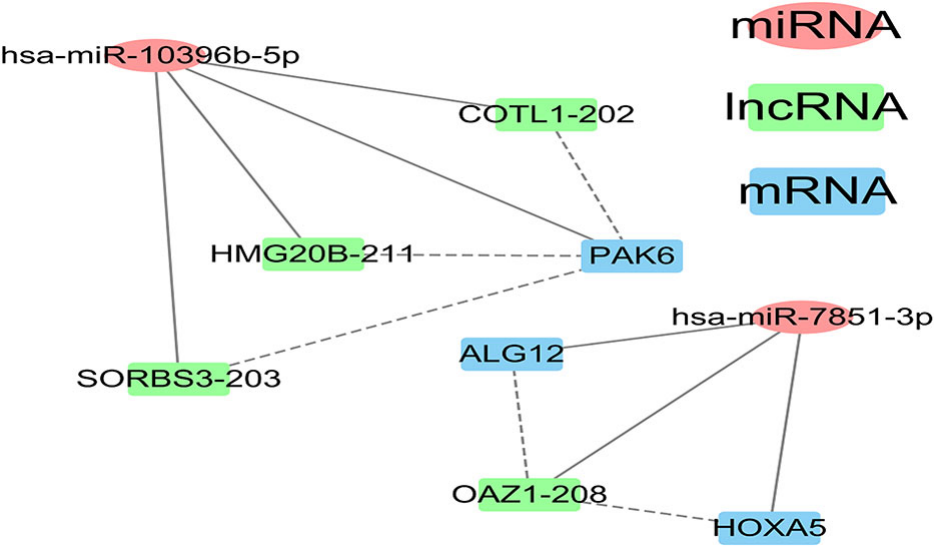
The intersection between the luminal B breast cancer and triple-negative breast cancer (TNBC) competing endogenous RNA (ceRNA) network of tissue exosomes derived from TNBC patients.

The intersected ceRNAs in tissue exosomes derived from luminal B BC and TNBC patients [lncRNA OAZ1/miRNA hsa-miR-7851-3p/multiple mRNAs (ALG12 and HOXA5), multiple lncRNAs (COTL1, HMG20B, SORBS3)/miRNA hsa-miR-10396b-5p/mRNA PAK6] are provided in **Figure 7**, and the relevant ceRNAs are listed in **Table 3**. Whether the intersected ceRNAs can be utilized to treat BC needs further detailed analysis. It was worth noting that PAK6 and hsa-miR-10396b-5p had more connectivity in TNBC than in luminal B BC (**Table 2**), indicating that a more complex ceRNA regulation might exist in TNBC than in luminal B BC.

## Discussion

The luminal B and TNBC subtypes of BC present distinct and disparate clinical behaviors and outcomes. Therefore, characterizing the complex ceRNA network will promote the understanding of heterogeneous BC and might provide new avenues to decipher new biomarkers and subtype-specific targets. Tissue exosomes derived from luminal B BC may regulate the RAS–MAPK pathway, and tissue exosomes derived from TNBC may regulate the development and growth of BC. In addition to ceRNA regulation that can predict patient survival, our results indicate that more complex regulation and heterogeneity might be involved in TNBC compared with that of luminal B BC.

After constructing and comparing the lncRNA–miRNA–mRNA ceRNA networks, we found nine common RNAs across the luminal B and TNBC subtypes, which play specific roles in these two BC subtypes. In luminal B BC, the regulation of lncRNA OAZ1/miRNA hsa-miR-7851-3p/multiple mRNAs (ALG12 and HOXA5) and multiple lncRNAs (COTL1, HMG20B, and SORBS3)/miRNA hsa-miR-10396b-5p/multiple mRNAs (PAK6, CEP41, RASA4) was found. While in TNBC, the regulation of lncRNAs (OAZ1, ARHGAP5)/miRNA hsa-miR-7851-3p/multiple mRNAs (ALG12, HOXA5, EVI5, and CYB5D2) and multiple lncRNAs (COTL1, HMG20B, SORBS3, RELA, PCMTD1, CBX7, ZFYVE21, and CENPV)/miRNA hsa-miR-10396b-5p/mRNAs (PAK6, ARVCF, CALCOCO1, CBX7, and SNUPN) was identified. Altogether, these results indicate that more complicated ceRNA regulation is indicated in TNBC. Among the nine common RNAs, miR-10396b-3p can inhibit collagen I and III by inhibiting interleukin-11 from alleviating ligamentum flavum hypertrophy induced by mechanical stress (18). In addition, hsa-miR-7851-3p can recognize highly conserved structures of the Coronaviridae family, while the other role of hsa-miR-7851-3p has not been deciphered. Moreover, the potential role of hsa-miR-7851-3p on HOXA6 and ALG12 identified in this study needs further analysis. This research illustrates that hsa-miR-10396b-5p and hsa-miR-7851-3p regulation is vital for the pathology of BC, and this information has not been reported previously.

In order to explore subtype-specific targets, we mainly focused on the specific RNAs in each subtype in this study. In luminal B BC, hsa-miR-1908-5p not only has specificity but also has more interactions. The relative expression of hsa-miR-1908-5p has been detected in BC tissues, adjacent normal tissues, BC patient serum, and healthy volunteer serum; therefore, it may exert oncogenic functions in cell differentiation, proliferation, metastasis, and invasion (19, 20). Furthermore, RAS signaling plays a vital role in the metastatic dissemination of luminal BC, which is associated with poor clinical outcomes (21). Additionally, MAPK/Fra1 pathway activation may alter the progenitor activity of the mammary gland to promote tumor progression (22). In this study, we found that hsa-miR-2277-5p, hsa-miR-10396b-5p, and hsa-miR-1273h-5p might be the potential miRNAs that regulate the activation of the RAS–MAPK pathway; however, more research is needed.

Endocrine system development and cancer growth-related ceRNA regulation are found in TNBC; *HOXA5, SCAPER, PAK6, PBX1, PITX1*, and *ALOX15B* are the relevant genes involved; and *hsa-miR-296-5p, hsa-miR-185-5p, hsa-miR-7851-3p, hsa-miR-3187-3p*, and *hsa-miR-10396b-5p* are the relevant miRNAs involved. With respect to the specific RNAs involved in TNBC, multiple miRNAs (*hsa-miR-3151-5p, hsa-miR-4446-3p*, and *hsa-miR-4763-3*p) are indicated to regulate the expression of WBP2, which may sustain BTRC mRNA stability to promote migration and invasion in TNBC *via* nuclear factor-κB activation (23).

Although the number of patients involved in this study was relatively small, the subtype-specific RNAs identified might promote the development of clinically approved exosome-based therapy. The analytical procedure we proposed might be a novel framework to decipher the function of tissue-derived exosomes in the tumor microenvironment based on the complex ceRNA network.

## Data availability statement

The raw sequence data reported in this paper have been deposited in the Genome Sequence Archive (Genomics, Proteomics & Bioinformatics 2021) in National Genomics Data Center (Nucleic Acids Res 2022), China National Center for Bioinformation / Beijing Institute of Genomics, Chinese Academy of Sciences (GSA-Human: HRA003927) that are publicly accessible at https://bigd.big.ac.cn/gsa-human/browse/HRA003927.

## Ethics statement

The studies involving human participants were reviewed and approved by Ethics Board of Yantai Yuhuangding Hospital. The patients/participants provided their written informed consent to participate in this study.

## Author contributions

JW, SY and DP conceived and designed the study. XZ and ZY collected data and conducted study. GQ and YM analyzed and interpreted the data. JW wrote the initial draft. DP revised the manuscript. JW had primary responsibility for the final content. All authors contributed to the article and approved the submitted version.

## Supplementary material

Supplementary Figure 1 Isolation of tissue exosomes from luminal B breast cancer and triple-negative breast cancer (TNBC) patients. **(A) **The bilayer membrane structure of exosomes was revealed with transmission electron microscopy. **(B)** The relative expression of exosome markers. CL, control; A276-06, persons with TNBC. **(C)** The size of each exosome was characterized using nanoparticle tracking analysis.

Supplementary Figure 2 MicroRNAs (miRNAs) involved in the competing endogenous RNA (ceRNA) network of luminal B breast cancer could predict the overall survival of breast cancer patients. The miRNAs involved in the ceRNA network of luminal B breast cancer were screened on miRpower with Kaplan–Meier analysis to obtain the miRNAs associated with patient survival.

Supplementary Figure 3 MicroRNAs (miRNAs) involved in the competing endogenous RNA (ceRNA) network of triple-negative breast cancer (TNBC) could predict the overall survival of breast cancer patients. The miRNAs involved in the ceRNA network of TNBC were screened on miRpower with Kaplan–Meier analysis to obtain the miRNAs associated with patient survival.

## References

[1] Sung H Ferlay J Siegel RL Laversanne M Soerjomataram I Jemal A. Global cancer statistics 2020: GLOBOCAN estimates of incidence and mortality worldwide for 36 cancers in 185 countries. CA Cancer J Clin (2021) 71:209–49. doi: 10.3322/caac.21660 33538338

[2] Kretzmann JA Irving KL Smith NM Evans CW. Modulating gene expression in breast cancer via DNA secondary structure and the CRISPR toolbox. NAR Cancer. (2021) 3:zcab048. doi: 10.1093/narcan/zcab048 34988459 PMC8693572

[3] Aguilar B Abdilleh K Acquaah-Mensah GK. Multi-omics inference of differential breast cancer-related transcriptional regulatory network gene hubs between young black and white patients. Cancer Genet (2023) 270-271:1–11. doi: 10.1016/j.cancergen.2022.11.001 36410105

[4] Johnson KS Conant EF Soo MS. Molecular subtypes of breast cancer: A review for breast radiologists. J Breast Imaging. (2020) 3:12–24. doi: 10.1093/jbi/wbaa110 38424845

[5] Dai X Cheng H Bai Z Li J. Breast cancer cell line classification and its relevance with breast tumor subtyping. J Cancer. (2017) 8:3131–41. doi: 10.7150/jca.18457 PMC5665029 29158785

[6] Demir Cetinkaya B Biray Avci C. Molecular perspective on targeted therapy in breast cancer: a review of current status. Med Oncol (2022) 39:149. doi: 10.1007/s12032-022-01749-1 35834030 PMC9281252

[7] Zhou X Liu J Wang W. Construction and investigation of breast-cancer-specific ceRNA network based on the mRNA and miRNA expression data. IET Syst Biol (2014) 8:96–103. doi: 10.1049/iet-syb.2013.0025 25014376 PMC8687191

[8] Abdollahzadeh R Daraei A Mansoori Y Sepahvand M Amoli MM Tavakkoly-Bazzaz J. Competing endogenous RNA (ceRNA) cross talk and language in ceRNA regulatory networks: A new look at hallmarks of breast cancer. J Cell Physiol (2019) 234:10080–100. doi: 10.1002/jcp.27941 30537129

[9] Lakshmi S Hughes TA Priya S. Exosomes and exosomal RNAs in breast cancer: A status update. Eur J Cancer. (2021) 144:252–68. doi: 10.1016/j.ejca.2020.11.033 33373870

[10] Kahlert C Kalluri R. Exosomes in tumor microenvironment influence cancer progression and metastasis. J Mol Med (Berl). (2013) 91:431–7. doi: 10.1007/s00109-013-1020-6 PMC4073669 23519402

[11] Bie N Yong T Wei Z Gan L Yang X. Extracellular vesicles for improved tumor accumulation and penetration. Adv Drug Delivery Rev (2022) 188:114450. doi: 10.1016/j.addr.2022.114450 35841955

[12] Li P Liu C Qian L Zheng Z Li C Lian Z. miR-10396b-3p inhibits mechanical stress-induced ligamentum flavum hypertrophy by targeting IL-11. FASEB J (2021) 35:e21676. doi: 10.1096/fj.202100169RR 34042220

[13] Liang R Han B Li Q Yuan Y Li J Sun D. Using RNA sequencing to identify putative competing endogenous RNAs (ceRNAs) potentially regulating fat metabolism in bovine liver. Sci Rep (2017) 7:6396. doi: 10.1038/s41598-017-06634-w 28743867 PMC5527063

[14] Bu D Luo H Huo P Wang Z Zhang S He Z. KOBAS-i: intelligent prioritization and exploratory visualization of biological functions for gene enrichment analysis. Nucleic Acids Res (2021) 49:W317–w25. doi: 10.1093/nar/gkab447 PMC8265193 34086934

[15] Zhou Y Zhou B Pache L Chang M Khodabakhshi AH Tanaseichuk O. Metascape provides a biologist-oriented resource for the analysis of systems-level datasets. Nat Commun (2019) 10:1523. doi: 10.1038/s41467-019-09234-6 30944313 PMC6447622

[16] Lánczky A Nagy Á Bottai G Munkácsy G Szabó A Santarpia L. miRpower: a web-tool to validate survival-associated miRNAs utilizing expression data from 2178 breast cancer patients. Breast Cancer Res Treat (2016) 160:439–46. doi: 10.1007/s10549-016-4013-7 27744485

[17] Yin L Duan JJ Bian XW Yu SC. Triple-negative breast cancer molecular subtyping and treatment progress. Breast Cancer Res (2020) 22:61. doi: 10.1186/s13058-020-01296-5 32517735 PMC7285581

[18] Li SR Man QW Gao X Lin H Wang J Su FC. Tissue-derived extracellular vesicles in cancers and non-cancer diseases: Present and future. J Extracell Vesicles. (2021) 10:e12175. doi: 10.1002/jev2.12175 34918479 PMC8678102

[19] Zhu Y Wang Q Xia Y Xiong X Weng S Ni H. Evaluation of MiR-1908-3p as a novel serum biomarker for breast cancer and analysis its oncogenic function and target genes. BMC Cancer. (2020) 20:644. doi: 10.1186/s12885-020-07125-4 32650755 PMC7350204

[20] Shen J Wu Y Ruan W Zhu F Duan S. miR-1908 dysregulation in human cancers. Front Oncol (2022) 12:857743. doi: 10.3389/fonc.2022.857743 35463352 PMC9021824

[21] Galiè M. RAS as supporting actor in breast cancer. Front Oncol (2019) 9:1199. doi: 10.3389/fonc.2019.01199 31781501 PMC6861383

[22] Godde NJ Sheridan JM Smith LK Pearson HB Britt KL Galea RC. Scribble modulates the MAPK/Fra1 pathway to disrupt luminal and ductal integrity and suppress tumour formation in the mammary gland. PloS Genet (2014) 10:e1004323. doi: 10.1371/journal.pgen.1004323 24852022 PMC4031063

[23] Lim YX Lin H Chu T Lim YP. WBP2 promotes BTRC mRNA stability to drive migration and invasion in triple-negative breast cancer *via* NF-κB activation. Mol Oncol (2022) 16:422–46. doi: 10.1002/1878-0261.13048 PMC8763649 34197030

