# Supplementary figures and images for: RNA atlas and competing endogenous RNA regulation in tissue-derived exosomes from luminal B and triple-negative breast cancer patients
Source: Front Oncol. 2023 Jul 7;13:1113115. doi: 10.3389/fonc.2023.1113115 (PMC10361514; doi:10.3389/fonc.2023.1113115)

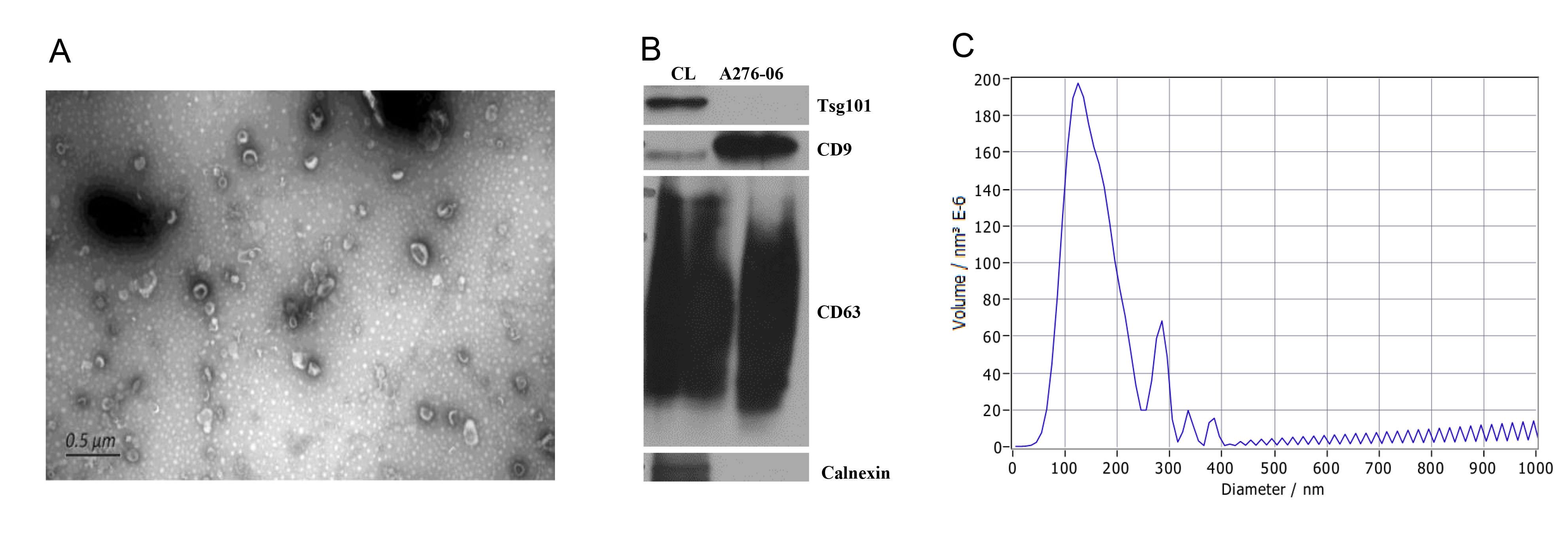

Supplement: Supplementary Figure 1 — Isolation of tissue exosomes from luminal B breast cancer and triple-negative breast cancer (TNBC) patients. (A) The bilayer membrane structure of exosomes was revealed with transmission electron microscopy. (B) The relative expression of exosome markers. CL, control; A276-06, persons with TNBC. (C) The size of each exosome was characterized using nanoparticle tracking analysis. [file Image_1.tif]

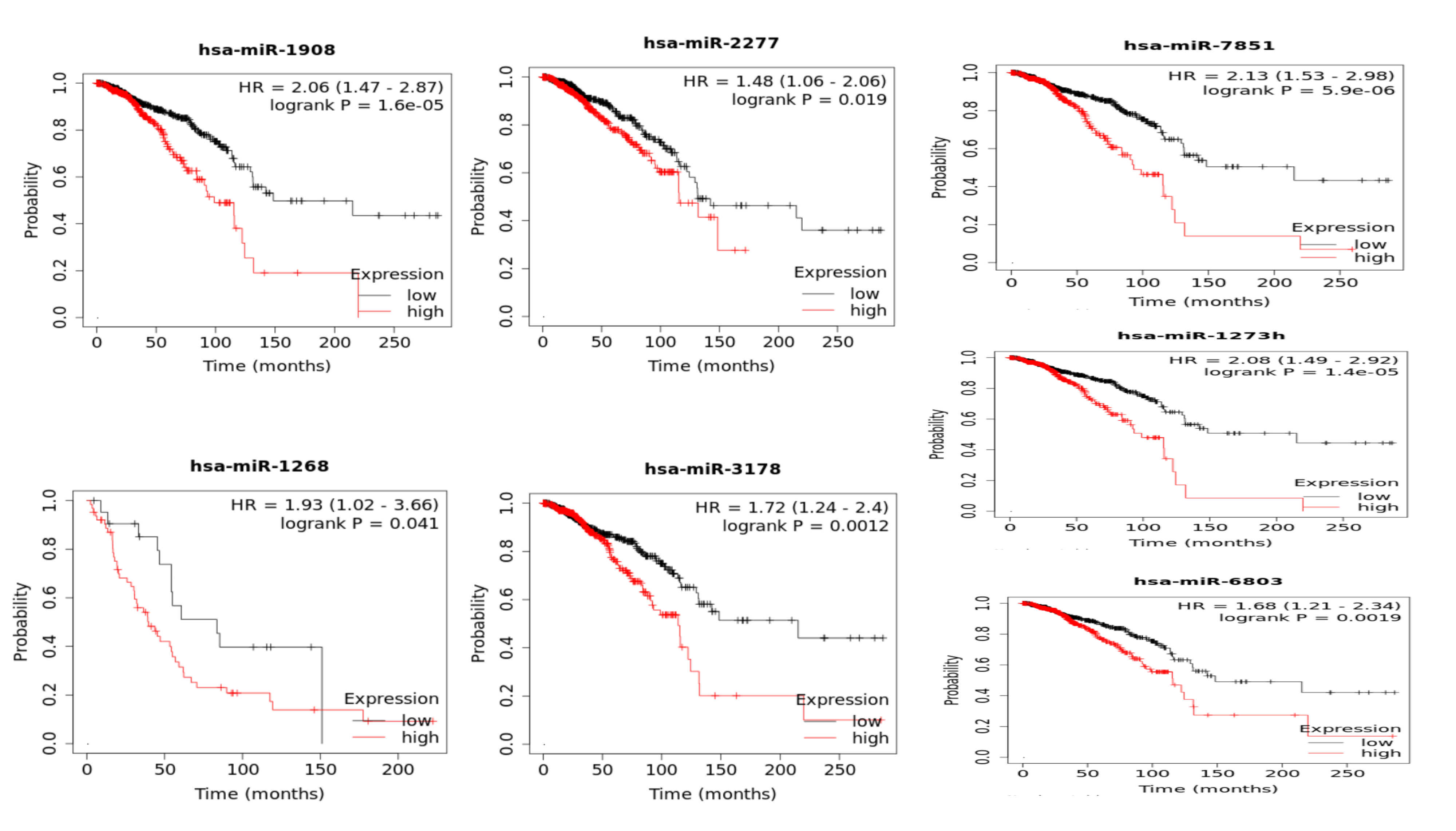

Supplement: Supplementary Figure 2 — MicroRNAs (miRNAs) involved in the competing endogenous RNA (ceRNA) network of luminal B breast cancer could predict the overall survival of breast cancer patients. The miRNAs involved in the ceRNA network of luminal B breast cancer were screened on miRpower with Kaplan–Meier analysis to obtain the miRNAs associated with patient survival. [file Image_2.tif]

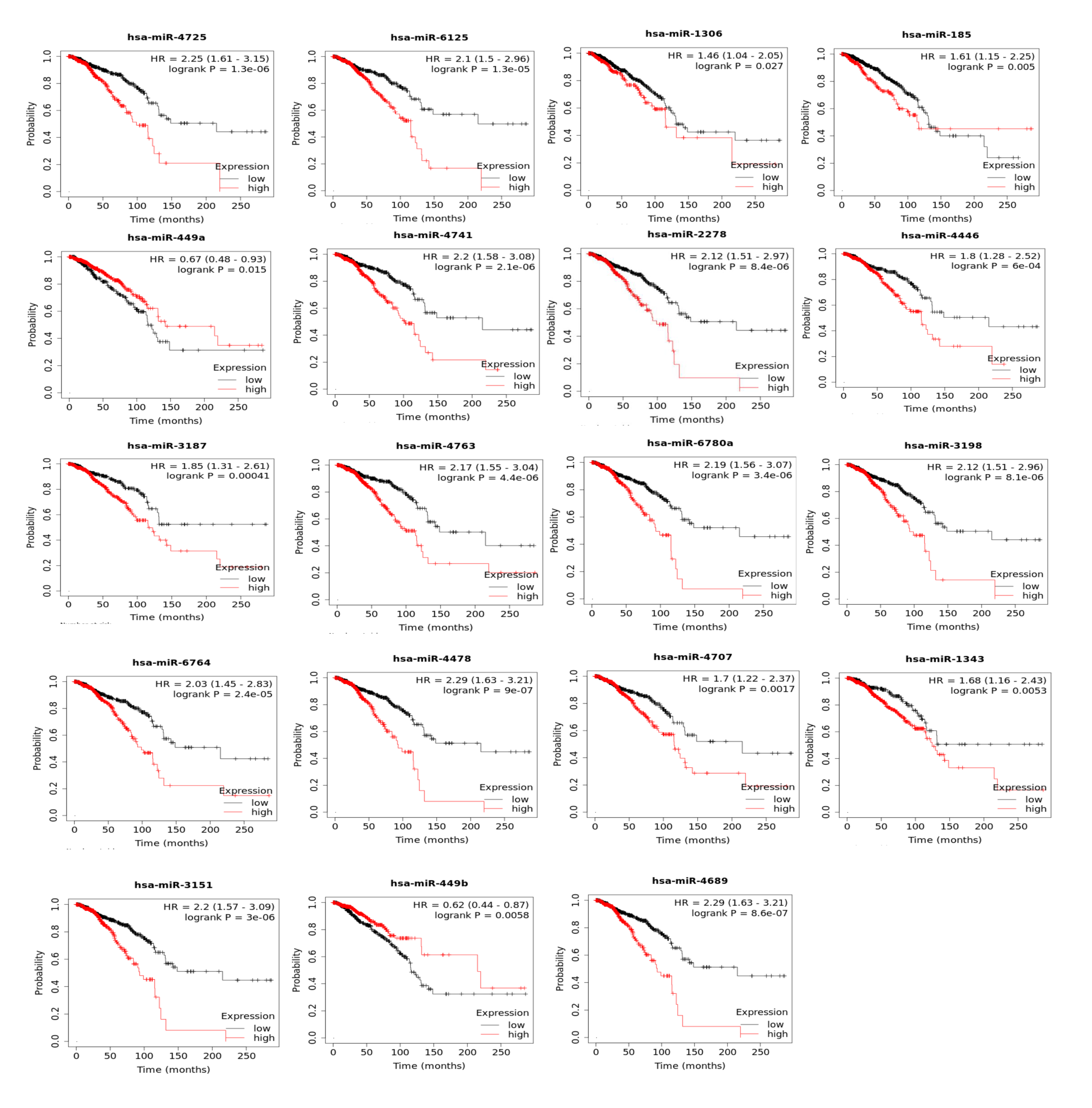

Supplement: Supplementary Figure 3 — MicroRNAs (miRNAs) involved in the competing endogenous RNA (ceRNA) network of triple-negative breast cancer (TNBC) could predict the overall survival of breast cancer patients. The miRNAs involved in the ceRNA network of TNBC were screened on miRpower with Kaplan–Meier analysis to obtain the miRNAs associated with patient survival. [file Image_3.tif]
